# Identification of Potential Cytokine Pathways for Therapeutic Intervention in Murine Primary Biliary Cirrhosis

**DOI:** 10.1371/journal.pone.0074225

**Published:** 2013-09-10

**Authors:** Kazuhito Kawata, Masanobu Tsuda, Guo-Xiang Yang, Weici Zhang, Hajime Tanaka, Koichi Tsuneyama, Patrick Leung, Xiao-Song He, Stuart Knechtle, Aftab A. Ansari, Ross L. Coppel, M. Eric Gershwin

**Affiliations:** 1 Division of Rheumatology, Allergy and Clinical Immunology, University of California at Davis School of Medicine, Davis, California, United States of America; 2 Hepatology Division, Department of Internal Medicine, Hamamatsu University School of Medicine, Shizuoka, Japan; 3 Department of Emergency and Critical Care Medicine, Kansai Medical University, Osaka, Japan; 4 Department of Diagnostic Pathology, Graduate School of Medicine and Pharmaceutical Science for Research, University of Toyama, Toyama, Japan; 5 Department of Surgery, The Emory Clinic and Hospital, Emory Transplant Center, Atlanta, Georgia, United States of America; 6 Department of Pathology, Emory University School of Medicine, Atlanta, Georgia, United States of America; 7 Department of Microbiology, Monash University, Clayton, Victoria, Australia; Escola Paulista de Medicina - UNIFESP, Brazil

## Abstract

Primary biliary cirrhosis (PBC) is considered a model autoimmune disease, with the most highly directed and specific autoantibody in both murine and human autoimmunity, the anti-mitochondrial autoantibody (AMA). However, therapeutic advances in this disease have lagged behind. Herein we have taken advantage of our unique model of murine PBC in which mice immunized with 2-octynoic acid coupled to BSA (2OA-BSA), a compound identified by quantitative structure activity relationships (QSAR) of human AMA binding, develop an intense inflammatory cholangitis with striking similarities to humans with PBC. In particular, we have constructed several unique gene-deleted mice, including mice deleted of IL-12p40, IL-12p35, IFN-γ, IL-23p19, IL-17A, IL-17F and IL-22, immunized these animals with 2OA-BSA and followed the natural history of immunopathology to identify key pathways that might provide clues for successful therapy. Our data indicate that whereas both IL-12/Th1 and IL-23/Th17 are involved in cholangitis, it is the IL-12/Th1 signaling pathway that elicits pathology. In fact, deletion of IFN-γ prevents disease and suppresses autoantibodies. Importantly, deletion of the Th17 cytokines IL-17A and IL-22, but not IL-17F, reduces biliary damage; IL-17A-knockout mice have reduced levels of anti-mitochondrial antibody. We further demonstrate that the production of IFN-γ is significantly decreased in the liver of IL-23p19^−/−^, IL-17A^−/−^ and IL-22^−/−^ mice compared with controls. However, the ability of T cells to produce IFN-γ was not affected in Th17 cytokine-deficient mice. Our data indicate that a deficient Th17 pathway suppresses the accumulation of IFN-γ producing cells in liver during the early phase of cholangitis. In conclusion, whereas IFN-γ has a pivotal role in the early events involved in the pathogenesis of autoimmune cholangitis induced by 2OA-BSA, the IL-23/Th17 pathway potentiates the effects of IL-12/IFN-γ-mediated immunopathology.

## Introduction

Primary biliary cirrhosis (PBC) is considered a Th1-dominant autoimmune response and Th1 effector cells are critical for the development of cholangitis [1], [2]. However, more recent data provide evidence that the Th17 effector cell response is also involved in disease pathogenesis [3], [4], [5]. Our group has reported that mice transgenic for directed expression of a dominant negative form of transforming growth factor beta receptor type II (dnTGF-βRII) spontaneously develop an autoimmune biliary ductular disease similar to human PBC, including development of anti-mitochondrial antibodies (AMA) [6]. However, deletion of IFN-γ did not protect these mice from autoimmune biliary disease [7]. Further deletion of the Th1 upstream signaling molecule, i.e. IL-12p35 subunit, in dnTGF-βRII mice, only delayed the appearance of inflammatory cell infiltrates in the liver and biliary cell damage [8]. Further, IL-12p35^−/−^ dnTGF-βRII mice demonstrated a distinct cytokine profile characterized by a shift from a prototype Th1 to a Th17 response associated with readily detectable occurrence of liver fibrosis, suggesting the involvement of a Th17 effector response in the development of biliary fibrosis [8]. However, it was reasoned that the mechanisms associated with biliary epithelial cell autoimmunity is complex and it remained unclear given the fact that depletion of IL-23 led to no marked pathologic changes in the liver of dnTGF-βRII mice [9].

Herein, we sought to examine the early involvement of the IL-12/Th1 pathway and IL-23/Th17 pathway in loss of tolerance in our well-defined xenobiotic-induced murine model of PBC in mice immunized with 2-octynoic acid coupled to bovine serum albumin (2OA-BSA) [10], [11], [12], [13]. We report herein that a deficiency in IL-12p40, but not IL-12p35 and IL-23p19, led to a completely abolition of autoimmune cholangitis. Strikingly, depletion of the prototypic Th1 cytokine IFN-γ, completely suppressed the development of cholangitis. In contrast, deletion of the major Th17 cytokine IL-17A or IL-22 did not inhibit development of portal inflammation and biliary cell damage. Our data indicate that while the IL-12/IFN-γ pathway is required for loss of tolerance, the IL-23/Th17 pathway potentiates IL-12/IFN-γ-mediated autoimmunity in this xenobiotic-induced cholangitis model. These findings further refine our understanding of the pathways that need to be targeted for therapy of human PBC.

## Materials and Methods

### Animals

Female wild-type (WT) C57BL/6J (B6), B6.129S7-IFN-γ^tm1Ts^ (IFN-γ^−/−^), B6.129S1-Il12a^tm1Jm^/J (IL-12p35^−/−^) mice and B6.129S1-Il12b^tm1Jm^ (IL-12p40^−/−^) mice were purchased from the Jackson Laboratory (Bar Harbor, ME). IL-23p19^−/−^, IL-17A^−/−^, IL-17F^−/−^, and IL-22^−/−^ mice were kindly obtained from Dr. Frederic J. de Sauvage (Genentech, South San Francisco, CA), Dr. Yoichiro Iwakura (University of Tokyo, Tokyo, Japan), Dr. Chen Dong (MD Anderson Cancer Center, Houston, TX), and Dr. Richard A. Flavell (Yale University, New Haven, CT), respectively. All mice were maintained in individually ventilated cages under specific pathogen-free conditions.

### Ethics Statement

Experiments were performed following approval from the University of California at Davis Animal Care and Use Committee.

### Experimental Protocol

Autoimmune cholangitis was induced as previously described [10], [11], [12]. Two-octynoic acid (2OA, a synthetic chemical mimic of lipoic acid-lysine located within the inner domain of PDC-E2) was coupled to bovine serum albumin (2OA-BSA). For the induction of autoimmune cholangitis, 100 µg 2OA-BSA conjugate (in 50 µl PBS) were emulsified with 50 µl of Complete Freund’s Adjuvant (CFA containing 1 mg/mL of Mycobacterium tuberculosis strain H37RA, Sigma-Aldrich, St. Louis, MO) and injected intraperitoneally (I.P.) into 8 week-old female B6 mice. The mice were subsequently boosted 2 weeks later with 2OA-BSA in incomplete Freund’s adjuvant (IFA, Sigma-Aldrich). Additionally, mice received 100 ng of pertussis toxin (List Biological Laboratories, Campbell, CA) on the same day and 2 days following the initial immunization with 2OA-BSA in CFA. Sera were collected at weeks 2, 4 and 8 after initial immunization with 2OA-BSA, and stored at °80°C until use. Mice were sacrificed 8 weeks after the initial immunization, and livers and spleens collected for histological evaluation, flow cytometric analysis for cellular phenotypes, and cell culture experiments as described below.

### Detection of Anti-PDC-E2 Antibodies (Abs)

Serum samples collected at different ages were tested for levels of anti-PDC-E2 antibodies using an enzyme-linked immunosorbent assay (ELISA). Briefly, 96-well ELISA plates were coated with 10 µg/ml of purified recombinant PDC-E2 in 100 µl of carbonate buffer (pH 9.6) at 4°C overnight, washed with Tris-Buffered Saline Tween-20 (TBS-T), and blocked with 5% skim milk in TBS for 30 minutes. One hundred µl of diluted sera (1∶250) were added to each well and incubated for 1 hour at room temperature. After washing, horseradish peroxidase (HRP)-conjugated anti-mouse immunoglobulin (A+M+G) (H+L) (1∶3,000) (Zymed, San Francisco, CA) was added. The plates were incubated for 1 hour at room temperature, the plates were re-washed and color developed with 100 µl of TMB peroxidase substrate (BD Biosciences, San Jose, CA) added to each well. Optical density (OD) was read at 450 nm. Previously calibrated positive and negative standards were included with each assay.

### Cell Culture and Cytokine Analysis

For analysis of secreted cytokines, an aliquot of 2.0×10^5^ intra-hepatic or splenic mononuclear cells (MNCs) were cultured in 96-well round-bottom plates in 200 µl of RPMI supplemented with 10% heat-inactivated fetal bovine serum (FBS) (GIBCO-Invitrogen Corp., Grand Island, NY), 100 µg/mL streptomycin, 100 U/mL penicillin, and 0.5 µg/mL each of anti-CD3 (BioLegend) and anti-CD28 monoclonal Abs (mAbs) (BioLegend). The cells were incubated for 72 hours at 37°C in a humidified 5% CO_2_ incubator. The supernatant fluid was collected for analysis. TNF-α, IFN-γ, IL-6, IL-2, IL-4, IL-10 and IL-17A were quantitated using a murine Th1/Th2/Th17 cytokine CBA kit (BD Biosciences, San Jose, CA). IL-17F and IL-22 were quantitated using ELISA MAX Deluxes (Biolegend).

### Flow Cytometry

MNCs were isolated from spleen and liver using density gradient centrifugation with Histopaque-1077 (Density: 1.077; Sigma-Aldrich, St Louis, MO). Anti-mouse CD16/32 mAb (clone 93, Biolegend) were used to block the Fc receptor prior to staining. MNCs were stained with fluorochrome-conjugated antibodies, including CD4, CD8α, NK1.1 (Biolegend, San Diego, CA), CD19 and TCRβ (eBioscience, San Diego, CA). Isotype Abs with matching conjugates were used as negative controls. Stained cells were analyzed using a FACScan flow cytometer (BD Bioscience) upgraded by Cytec Development (Fremont, CA) to allow for five-color analysis. Data were analyzed using CELLQUEST software (BD Bioscience).

### Histopathology and Immunohistochemistry

Liver tissues were fixed in 4% paraformaldehyde at room temperature for 2 days, embedded in paraffin, and cut into 4-µm sections. The sections were deparaffinized, stained with hematoxylin and eosin (H&E). Liver disease staging was performed and evaluated using our previously defined indices by a “blinded” pathologist [14]. Monoclonal antibodies against cell surface markers, including CD4 (BioLegend, San Diego, CA) and CD8α (BioLegend) were used for immunohistochemical staining of liver sections as described [6].

### Statistical Analysis

The data reflect mean ± SEM. Two-sample comparisons were carried out with a two-tailed unpaired Mann-Whitney test. One-way ANOVA was performed for differences among the three groups followed by Kruskal-Wallis and Dunn’s multiple comparisons test. A value of p<0.05 was considered statistically significant. The number of mice in each protocol is noted in the figure legends.

## Results

### The IL-12/IL-23 p40 Signaling Pathway is Essential for the 2OA-BSA-induced Hepatic Inflammatory Response

**Figure 1:**
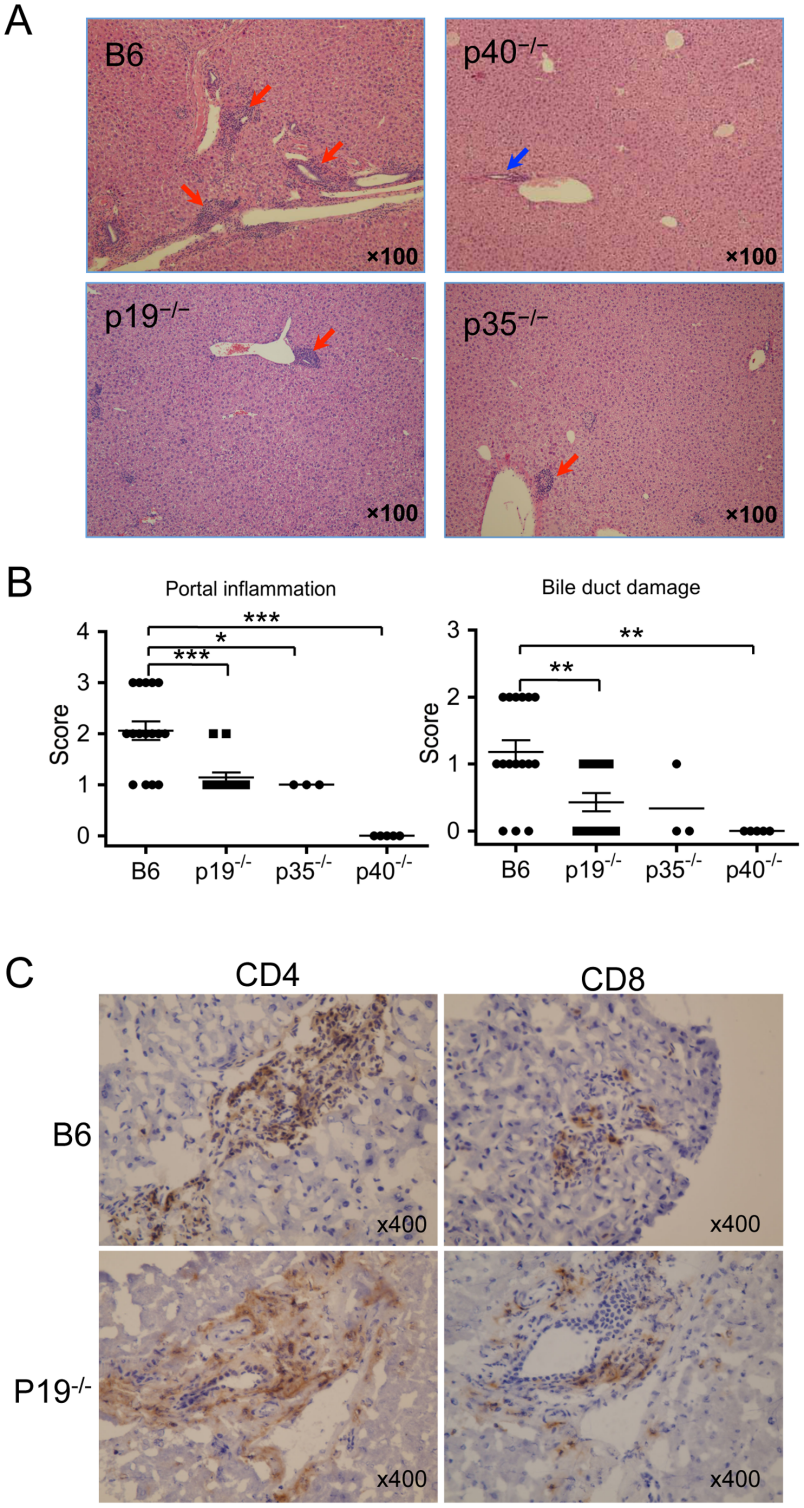
IL-12p40 is required for 2OA-BSA-induced cholangitis. (A) H&E staining of representative liver from WT, IL-12p40^−/−^ (p40^−/−^), IL-23^−/−^ (p19^−/−^) and IL-12p35^−/−^ (p35^−/−^) mice 8 weeks after 2OA-BSA immunization. Portal inflammatory changes with interlobular bile duct damage (red arrow) were observed in the liver of WT, p19^−/−^ and p35^−/−^ mice but not p40^−/−^ mice; normal bile ducts in p40^−/−^ mice (blue arrow). (B) Portal inflammation and bile duct damage were examined in individual animals. The pathological score of portal inflammation and biliary cell damage were evaluated in WT (n = 17), p19^−/−^ (n = 14), p35^−/−^ (n = 3) and p40^−/−^ (n = 5) mice. *p<0.05, **p<0.01, ***p<0.001. (C) Immunohistochemical analysis of the liver of WT mice and IL-23p19^−/−^ mice at 8 weeks after 2OA-BSA immunization using mAbs to CD4 and CD8.

**Figure 2:**
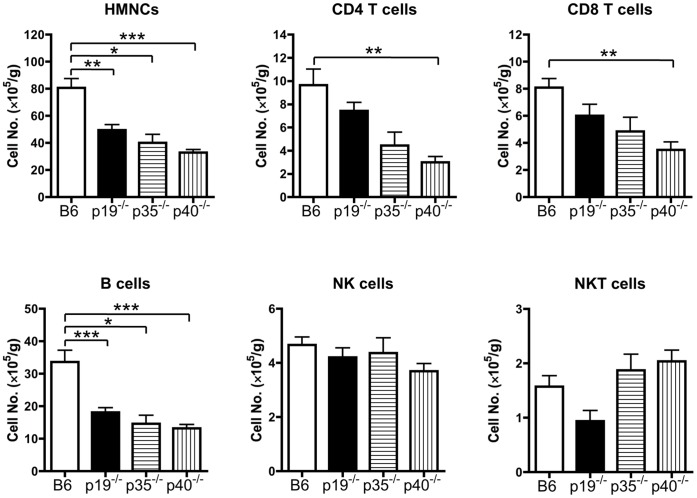
Flow cytometric analysis of the lymphocytic infiltration in liver from IL-23p19^−/−^, IL-12p35^−/−^ and control WT mice. HMNCs were isolated from liver samples at 8 weeks after the first immunization. The data are expressed as the cell number per gram of tissue. **p<0.01, ***p<0.001.

To address the requirement of the Th1/Th17 signaling pathway in loss of tolerance, autoimmune cholangitis was induced by 2OA-BSA immunization. We initially examined liver immunopathology in p19^−/−^ (Th17-deficient), p35^−/−^ (Th1-deficient) and p40^−/−^ (both Th1- and Th17-deficient) mice, in comparison with WT mice on the same B6 background after immunization with 2OA-BSA. Eight weeks after immunization, while there was readily detectable portal mononuclear cell infiltration in the p19^−/−^ and p35^−/−^ mice, no detectable lymphocytic infiltration was noted in the p40^−/−^ mice. The liver of p19^−/−^ and p35^−/−^ mice, however, demonstrated less portal inflammation and biliary cell damage compared with WT mice (Figure 1A and 1B). Immunohistochemical analysis was carried out in efforts to address the levels of infiltrating CD4^+^ and CD8^+^ T cells in portal tracts. These studies indicate the presence of both CD4^+^ and CD8^+^ T cells infiltrate in affected portal tracts but there was no detectable difference in the phenotype of cellular infiltrates between the WT and IL-23p19^−/−^ mice (Figure 1C). We further analyzed the phenotype of intra-hepatic mononuclear cells (HMNCs). While the total numbers of MNCs and B cells were significantly decreased in p19^−/−^, p35^−/−^ and p40^−/−^ mice, the number of intra-hepatic CD4 and CD8 T cells were significantly decreased only in p40^−/−^ mice as compared with WT mice (Figure 2). These data indicate that the Th1 and Th17 signaling pathways are both involved in the appearance of autoimmune cholangitis.

**Figure 3:**
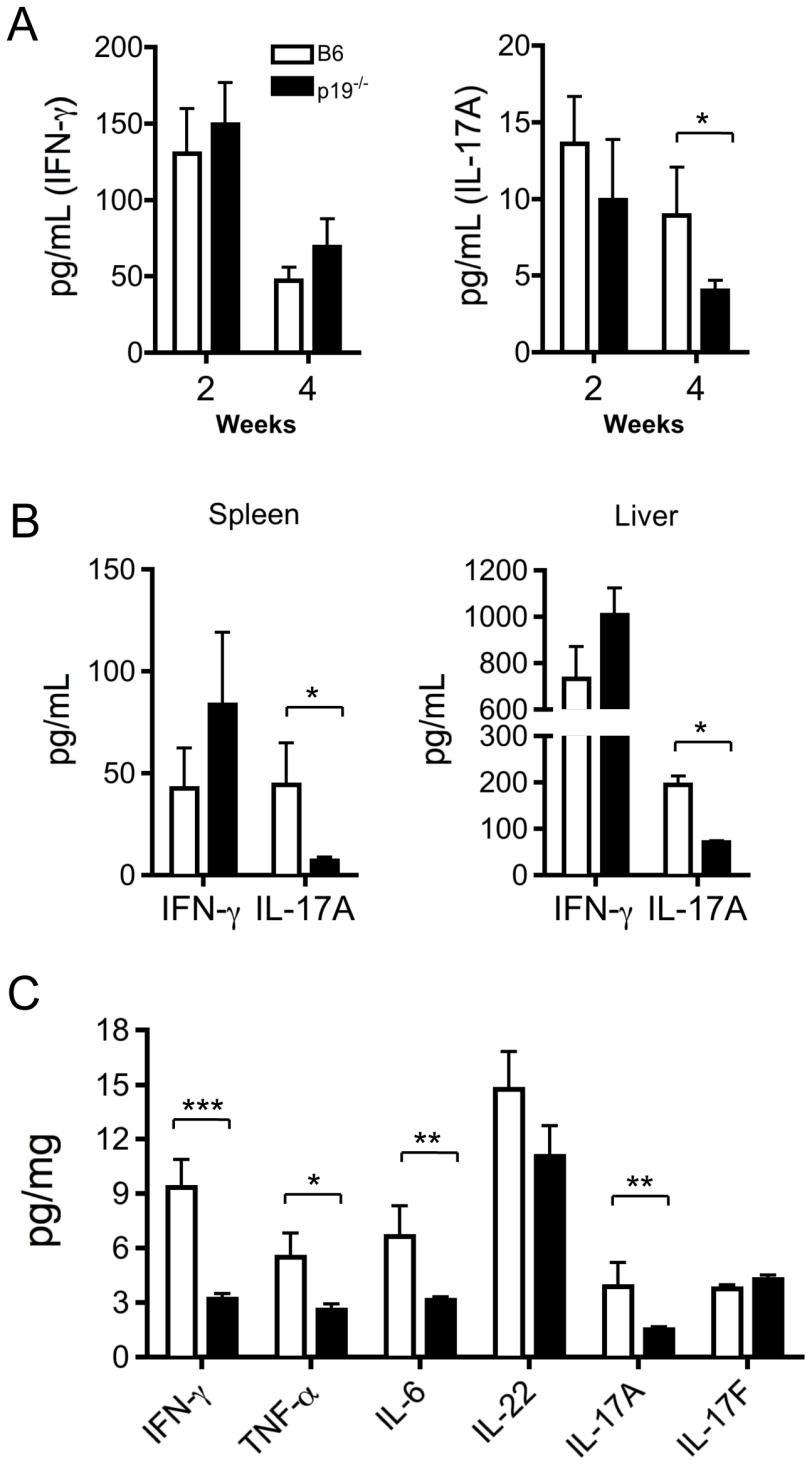
Inflammatory cytokine production in mice immunized with 2OA-BSA. (A) Serum levels of IFN-γ and IL-17A in B6 and IL-23p19^−/−^ mice 2 and 4 weeks after initial 2OA-BSA immunization. (B) Production of IFN-γ and IL-17A in supernatant fluids of cultured splenic and hepatic MNCs (n = 4) with anti-CD3/CD28 mAbs for 3 days (C) inflammatory cytokines in extracted liver protein from WT mice (n = 8) and IL-23p19^−/−^ (n = 8) mice. *p<0.05, **p<0.01, ***p<0.001.

We next measured the production of the representative Th1/Th17 cytokines IFN-γ and IL-17A in the sera of p19^−/−^ mice after 2OA-BSA immunization. As shown in Figure 3A, while sera from the 2OA-BSA-immunized IL-23p19^−/−^ mice had decreased levels of IL-17A, levels of IFN-γ were not influenced by lack of IL-23. We then stimulated MNCs with anti-CD3/CD28. Similar to the levels of these cytokines in peripheral blood, while TCR activation did not induce detectable change in the levels of IFN-γ, the levels of IL-17A production by either spleen or liver T cells were markedly decreased. This indicates that the IFN-γ-producing ability was not affected by deleting p19. However, as shown in Figure 3C, the amount of IFN-γ in liver extracted protein was significantly decreased in 2OA-BSA-immunized p19^−/−^ mice. These data suggest that deficiency in the Th17 signaling pathway may also suppress the synthesis of IFN-γ by biliary infiltrating cells.

### Genetic Deletion of IL-17A and IL-22 Prevented 2OA-BSA-induced Cholangitis in Mice

**Figure 4:**
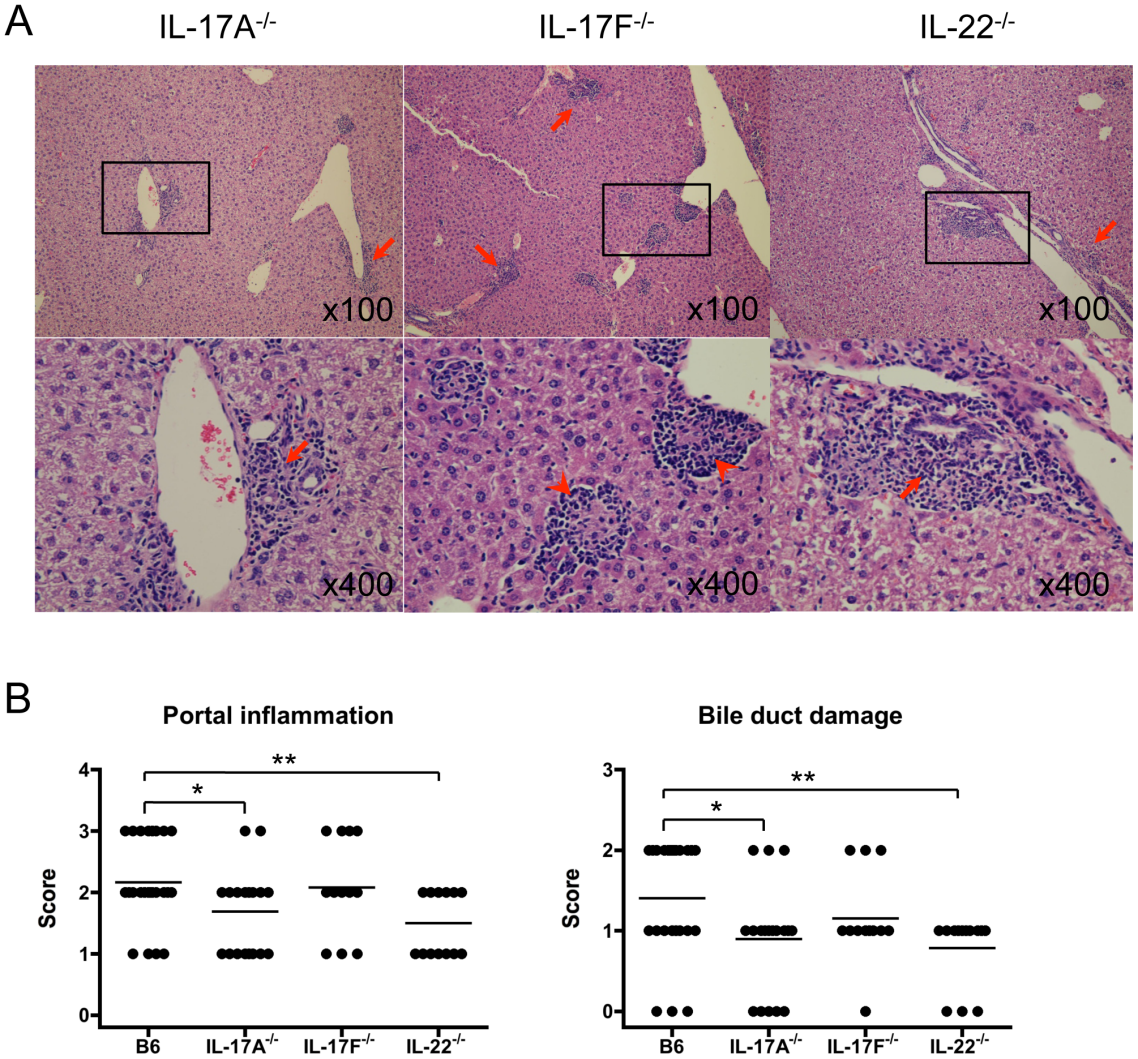
Pathological changes in liver of mice immunized with 2OA-BSA. (A) Representative H&E staining profiles of liver from IL-17A^−/−^, IL-17F^−/−^ and IL-22^−/−^ mice 8 weeks after immunization. Portal inflammatory changes with interlobular bile duct damage (red arrow) were observed. IL-17F^−/−^ demonstrating more epithelioid granulomas (red arrowhead) in liver compared to IL-17A^−/−^ and IL-22^−/−^ mice. (B) Scoring of portal inflammation and bile duct damage in liver from WT (n = 25), IL-17A^−/−^ (n = 19), IL-17F^−/−^ (n = 13) and IL-22^−/−^ (n = 14) mice.

To address the contribution of the IL-23/Th17-dependent pathway in 2OA-BSA-induced autoimmune cholangitis, we further compared the liver pathology of IL-17A^−/−^, IL-17F^−/−^ and IL-22^−/−^ mice with WT mice. As expected, WT B6 mice developed cholangitis exemplified by portal lymphocytic infiltrations with bile duct damage and granulomatous destruction at 8 weeks. In contrast, the cholangitis in IL-17A^−/−^ and IL-22^−/−^ mice was significantly attenuated as evaluated by portal inflammation and bile duct damage (Figure 4). In addition, histopathological scoring did not reveal obvious differences in the levels of cholangitis in IL-17F^−/−^ mice compared to controls. These data indicate that the influence of IL-23 is via IL-17A and/or IL-22, but not IL-17F, to regulate the level of inflammation and the severity of cholangitis induced by 2OA-BSA immunization.

**Figure 5:**
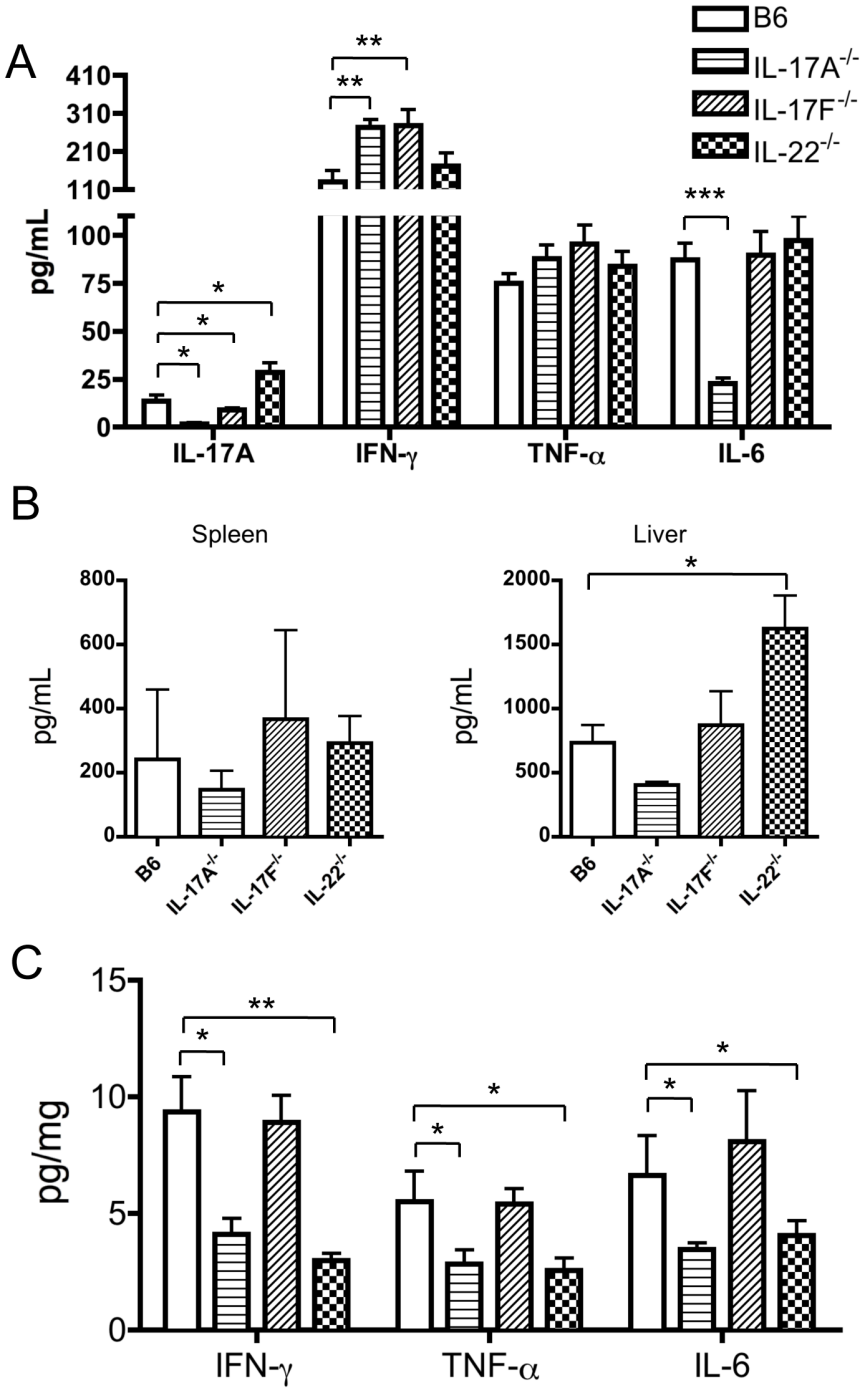
Inflammatory cytokine production in mice immunized with 2OA-BSA. (A) Serum levels of IL-17A, IFN-γ, TNF-α and IL-6 in WT, IL-17A^−/−^, IL-17F^−/−^ and IL-22^−/−^ mice, respectively, at 2 weeks following 2OA-BSA immunization. (B) Production of IFN-γ in supernatant fluids of cultured splenic and hepatic MNCs (n = 4) with anti-CD3/CD28 mAbs at 3 days. (C) The level of inflammatory cytokines in extracted liver protein from WT, IL-17A^−/−^, IL-17F^−/−^ and IL-22^−/−^ mice, respectively. Each group n = 8. *p<0.05, **p<0.01, ***p<0.001.

We then analyzed the levels of serum IFN-γ in these Th17-deficient mice during the induction of cholangitis. As shown in Figure 5A, the level of IFN-γ was higher in IL-17A^−/−^ and IL-17F^−/−^ mice but not in IL-22^−/−^ mice compared to WT mice. In contrast, IL-17A production was increased in IL-22^−/−^ mice 2 weeks. In addition, low levels of serum IL-6 were only found in IL-17A^−/−^ mice. In efforts to address the ability of liver infiltrated T cells to synthesize IFN-γ, fresh isolated MNCs from mouse spleen and liver were cultured with anti-CD3/CD28 antibodies for 3 days and the supernatants analyzed. The data in Figure 5B demonstrate no detectable changes in the level of IFN-γ synthesized by both splenic and hepatic MNCs isolated from both IL-17A^−/−^ and IL-17F^−/−^ mice, as compared with that of controls. Only liver MNCs from IL-22^−/−^ mice produced more IFN-γ than that of B6 mice after TCR activation. Of note, the inflammatory cytokines IFN-γ, TNF-α and IL-6 in extracted liver protein were significantly decreased in IL-17A^−/−^ and IL-22^−/−^ mice, but not IL-17F^−/−^ mice, which is consistent with the extent of liver inflammation. These data suggest that the Th17 cytokine IL-17A or IL-22 may promote the influx and/or the accumulation of inflammatory Th1 cells into liver during the development of cholangitis.

### Key Role of IFN-γ in the Induction of Cholangitis by 2OA-BSA Immunization

**Figure 6:**
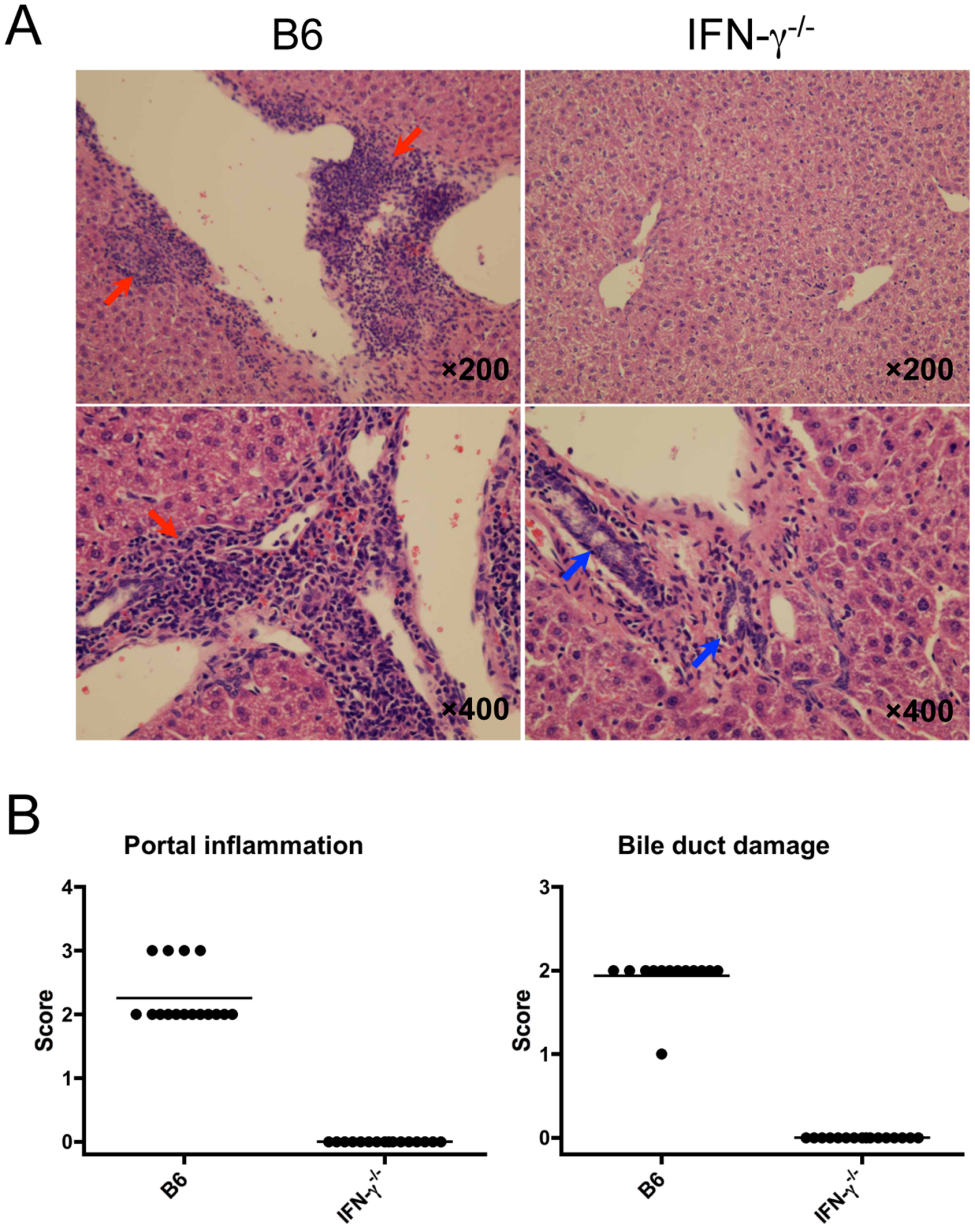
Pathological changes in the liver of IFN-γ^−/−^ mice immunized with 2OA-BSA. (A) Representative H&E stained of liver sections from IFN-γ^−/−^ mice compared with WT mice 8 weeks after 2OA-BSA immunization. Portal inflammatory changes with interlobular bile duct damage (red arrow) were observed in WT mice; normal bile ducts in IFN-γ^−/−^ mice (blue arrows). (B) Scoring of portal inflammation and bile duct damage in sections of liver from WT and IFN-γ^−/−^ mice (each group, n = 16).

**Figure 7:**
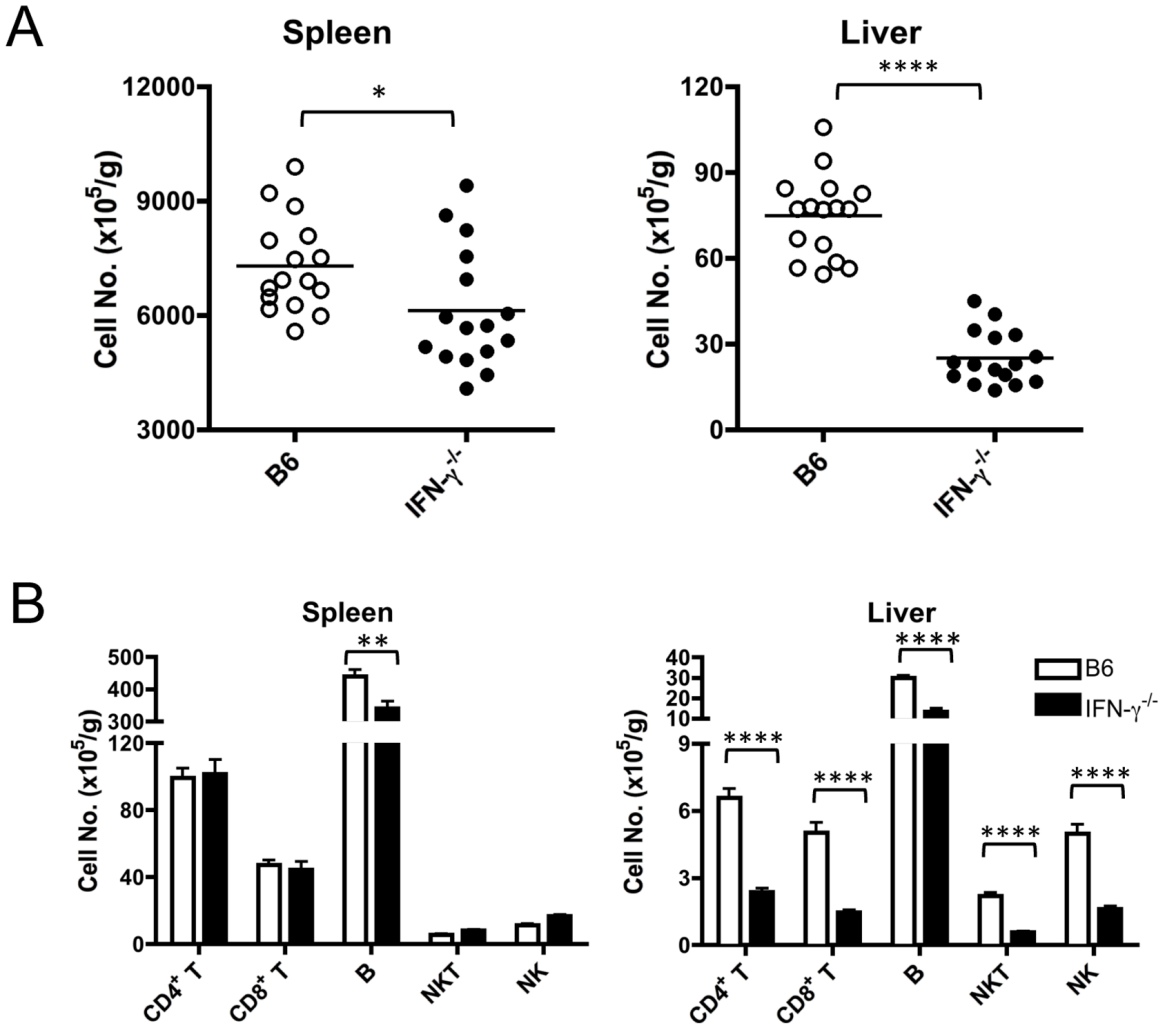
Genetic deletion of IFN-γ results in decreased lymphocytic infiltration of the liver. MNCs were isolated from spleen and liver samples of WT and IFN-γ^−/−^ mice 8 weeks after 2OA-BSA immunization. The number of MNCs and cell subpopulations were analyzed by standard flow cytometry. The data are expressed as the cell number per gram of tissue. Each group, n = 16; * p<0.05; ***p<0.001; ****p<0.0001.

Since the occurrence of cholangitis was completely abrogated only in p40^−/−^ mice but not in Th17-deficient mice (p19^−/−^, IL-17A^−/−^ and IL-22^−/−^) and Th1-deficient p35^−/−^ mice, we next attempted to determine the role of IFN-γ in the induction of cholangitis. Thus, the IFN-γ^−/−^ mice were immunized with 2OA-BSA and cholangitis was compared with that of WT mice. Very strikingly, there was no obviously lymphocytic infiltrates in the liver of IFN-γ^−/−^ mice 8 weeks after 2OA-BSA immunization (Figure 6), similar to their absence in the p40^−/−^ mice. We further counted and analyzed the phenotype of infiltrating cells in the liver of IFN-γ^−/−^ mice, which included CD4^+^ T cells, CD8^+^ T cells, B cells, NKT cells and NK cells. These studies demonstrate a significant decrease of total MNC number in both liver and spleen of 2OA-BSA-immunized IFN-γ^−/−^ mice as compared with controls (Figure 7). Flow cytometry analysis revealed that the number of hepatic CD4^+^ T cells, CD8^+^ T cells, B cells, NKT cells and NK cells were significantly reduced in IFN-γ^−/−^ mice (Figure 7). These data indicate that IFN-γ has a key role in the early events that lead to the development of 2OA-BSA-induced cholangitis.

### Role of Th1 vs Th17 Signaling in the Production of AMA in 2OA-BSA-immunized Mice

**Figure 8:**
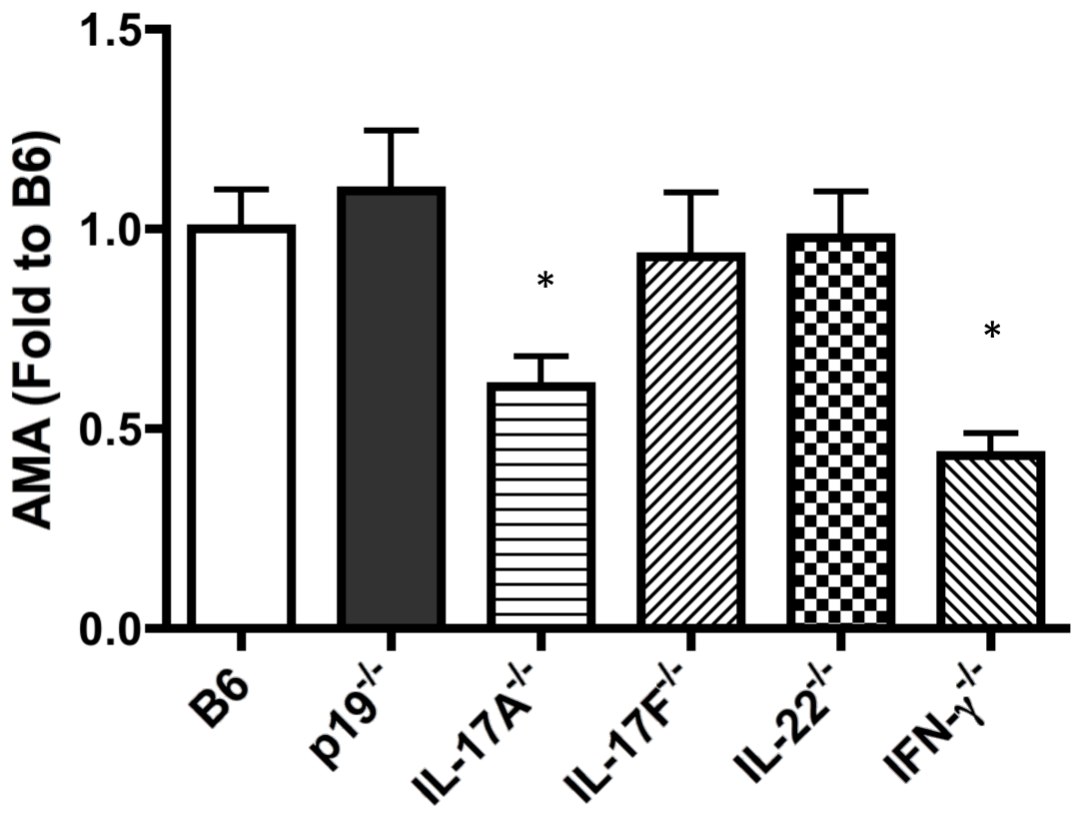
Comparative levels of PDC-E2-specific autoantibodies in cytokine deficient mice induced by 2OA-BSA immunization; samples were collected at serial time points. The fold changes of AMA OD values of each group are compared to the data of WT mice (n = 8–16/group). AMA values of IL-17A^−/−^ and IFN-γ mice compared to other groups, *p<0.05.

To compare the role of cytokine deletion in the induction of AMA, levels of serum anti-PDC-E2 antibodies were measured 8 weeks following 2OA-BSA immunization. As shown in Figure 8, significantly lower levels of anti-PDC-E2 antibodies were detected in the sera from the IFN-γ^−/−^ and IL-17A^−/−^ mice, but not in IL-22^−/−^, IL-17F^−/−^ and IL-23p19^−/−^ mice. Of note, although the extent of cholangitis induced by 2OA-BSA immunization is different as shown in Figures 4 and 6, AMA production in IFN-γ^−/−^ was not significantly reduced compared to the data in IL-17A^−/−^ mice. These data suggest that while both IL-17A and IFN-γ are important for AMA production, IFN-γ appears to play a more prominent role in the induction of cholangitis.

## Discussion

IL-12-mediated IFN-γ and Th1 responses have been implicated in the pathogenesis of various autoimmune diseases [15], [16], [17]. However, the requirement for IL-12/IFN-γ signaling has recently been questioned by several studies in animal models, in which the IL-23/IL-17 pathway was found to be critical for the induction of autoimmune inflammation [16], [18], [19], [20], [21]. Thus, the role of Th1 versus Th17 in the pathogenesis of PBC remains unresolved. The studies conducted herein were aimed to address this unresolved issue. Of interest, the results of the studies reported herein demonstrate that a critical role exists for both the IL-12/IFN-γ and IL-23/IL-17 signaling pathways in mediating cholangitis in mice induced by xenobiotic immunization. The data demonstrate that depletion of p40 in mice, which decreases both the IL-12/Th1 and IL-23/Th17 signaling pathway, completely prevented the development of cholangitis, including portal inflammatory cell infiltrates and biliary epithelial cell damage. On the other hand mice genetically deficient in either IL-12p35 or IL-23p19 only partially suppressed liver disease. These data indicate that both Th1 and Th17 effector responses are involved in autoimmunity to biliary epithelial cells. Deletion of either IL-12p35 or IL-23p19 is not sufficient to prevent the development of cholangitis induced by 2OA-BSA immunization.

It is of interest that deletion of IFN-γ in 2OA-BSA-immunized mice completely suppressed the occurrence of inflammatory cells infiltrates in liver associated with prevention of bile duct damage. However, deletion of the IL-23p35 chain in mice, which is the upstream cytokine for inducing a Th1/IFN-γ effector response, still developed cholangitis despite the fact that disease severity is strikingly decreased. The cytokine IL-12 is comprised of IL-12p35 and IL-12p40 subunits and is critical for the induction of IFN-γ from T and NKT cells [22] **.** The heterodimer p19/p40, known as IL-23, a member of the IL-12 family cytokine, also regulates Th1-cell responses [23], [24]. Thus, the p40 protein should be considered to be an IL-12–specific subunit required for the functional expression of IL-12. IL-23 production and the IL-23/Th17 pathway are unaffected in p35^−/−^ mice despite an impairment in the expression of IFN-γ, whereas the IL-17A/R signal is upstream of IFN-γ in the mediation of inflammation [25]. Also, administration of an IL-23 plasmid to IL-12p40^−/−^ mice up-regulates production of IFN-γ in response to Mycobacterium tuberculosis [26] and IL-23 promotes IFN-γ production, rather than IL-17 production, via an IL-23– and TNF-α–dependent pathway in human inflammatory bowel disease [27]. These findings and our current results indicate that IL-23 is involved in the pathogenesis of disease not only through IL-23/IL-17, but also through a previously undefined IL-23/IFN-γ axis. Finally, the IL-23 induced Th1 effector response, i.e. IFN-γ production, may be required in eliciting biliary cell autoimmunity in p35^−/−^ mice.

The IL-23/Th17 pathway plays a key role in the murine model of T cell-mediated autoimmune encephalomyelitis and other autoimmune disease [18], [28], [29], [30], [31]. Thus, to examine the role of the IL-23/Th17 pathway in the pathogenesis of cholangitis induced herein, we first investigated IL-23p19^−/−^ mice. IL-23 shares its p40 subunit with IL-12 but has a unique p19 subunit [32] and is a critical cytokine controlling the development of Th17 cells. We demonstrate that portal inflammation and bile duct damage in IL-23p19^−/−^ mice were ameliorated compared with controls. Furthermore, inflammatory MNCs infiltration and inflammatory cytokine production were markedly decreased in the liver of IL-23p19^−/−^ mice. We reason that this is due to the deficient differentiation of Th17 cells consistent with the observation of significantly decreased levels of IL-17A and IL-22 in IL-23p19^−/−^ mice. We therefore compared the role of IL-17A, IL-17F and IL-22, which are the major Th17 related cytokines. Portal inflammation and biliary cell damage were significantly suppressed in IL-17A^−/−^ and IL-22^−/−^, but not in IL-17F^−/−^, as compared to WT mice. However, the liver histopathology of IL17F^−/−^ mice mimic that of IL-17A^−/−^ and IL-22^−/−^ mice. Given that all three knockout mice have a similar degree of cholangitis, blockade of a single Th17 cytokine is insufficient to completely suppress the liver immunopathology. We note that lack of IL-17A (but not IL-17F and IL-22) inhibits serum AMA production (Figure 8). Therefore, we reason that the role of IL-23 in biliary autoimmunity was due to its ability to promote differentiation of T cells to a distinct effector subtype producing IL-17A and IL-22. Future studies on the interactions of these cytokines should be addressed in order to focus on potential therapeutic targets in humans with PBC.

Our data should be compared with observations in other autoimmune diseases which have suggested a requirement for IL-23 rather than IL-12 for disease induction by active immunization with the appropriate autoantigen mixed in CFA. IL-12p35-deficient mice are susceptible to experimental autoimmune encephalomyelitis (EAE), whereas the disease was suppressed in IL-17^−/−^ and IL-23p19^−/−^ mice [33], [34], [35]. The development of EAE is exacerbated in IFN-γ^−/−^ and/or IFN-γR^−/−^ mice [36], [37], indicating that IFN-γ serves a protective role. Using knockout mice, IL-23p19 is essential for the induction of experimental autoimmune uveitis (EAU) and a pro-inflammatory cytokine response [20]. Both IL-12p35^−/−^ and IFN-γ^−/−^ mice characteristically develop more severe inflammation and retinal damage compared with controls [20]. It should be noted that the deletion of IFN-γ in dnTGF-βRII mice, which spontaneously developed a PBC-like disease, did not suppress either portal inflammation or biliary cell damage in their livers [7]. Given the fact that the T cells were activated due to a deficiency in the TGF-β signaling pathway in the dnTGF-βRII mice [38], the present studies suggest that IFN-γ might be an initial trigger during the development of human PBC.

IL-22 is thought to serve as a double-edged sword. Thus, IL-22 plays not only an inflammatory role but also a protective role in autoimmune diseases. This view is supported by a previous report that IL-22^−/−^ mice were less susceptible to collagen-induced arthritis than wild type mice [39]. In contrast, an IL-22 gene-delivery system ameliorates intestinal inflammation in a mouse model of ulcerative colitis [40]. In the studies reported herein, we further demonstrate that IL-22^−/−^ tends to decrease cholangitis. The liver pathological changes were comparable to that noted in IL-17A^−/−^ mice. Since the expression of IL-17A and IFN-γ in T cells was not suppressed in IL-22^−/−^ mice and IFN-γ production by intra-hepatic T cells was even increased by activation via TCR, the mechanism of disease suppression in the IL-22-deficient mice needs to be studied in future studies. In conclusion, our data demonstrated a strict requirement of the activation of the IL-12/Th1 pathway, especially the IFN-γ signaling pathway. Although IFN-γ is considered as the initial trigger during the breaking of tolerance, both Th1 and Th17 effector responses and/or their interaction contribute to the ongoing pathology. We have previously postulated that PBC is a multi-hit process in which both the innate and adaptive immune systems will contribute to pathology at different stages of the natural history of disease. Clearly, dissection of these pathways may end up with a personalized medicine approach for treatment of patients and, in particular, the understanding of events that initially lead to breach of tolerance versus those adaptive continued responses that result in biliary pathology and ultimately fibrosis.
